# Recombination Located over 2A-2B Junction Ribosome Frameshifting Region of Saffold Cardiovirus

**DOI:** 10.3390/v10100520

**Published:** 2018-09-24

**Authors:** Antônio Charlys da Costa, Adriana Luchs, Flávio Augusto de Pádua Milagres, Shirley Vasconcelos Komninakis, Danielle Elise Gill, Márcia Cristina Alves Brito Sayão Lobato, Rafael Brustulin, Rogério Togisaki das Chagas, Maria de Fátima Neves dos Santos Abrão, Cassia Vitória de Deus Alves Soares, Xutao Deng, Ester Cerdeira Sabino, Eric Delwart, Élcio Leal

**Affiliations:** 1 Institute of Tropical Medicine, University of São Paulo, São Paulo 05403-000, Brazil; degill@g.clemson.edu (D.E.G.); sabinoec@gmail.com (E.C.S.); 2 Enteric Disease Laboratory, Virology Center, Adolfo Lutz Institute, São Paulo 01246-000, Brazil; driluchs@gmail.com; 3 LIM/46, Faculty of Medicine, University of São Paulo, São Paulo 01246-903, Brazil; flaviomilagres@uft.edu.br; 4 Secretary of Health of Tocantins, Tocantins 77453-000, Brazil; eumarciaalvesbrito@gmail.com (M.C.A.B.S.L.); eu3rafael@gmail.com (R.B.); chagastogisaki@hotmail.com (R.T.d.C.); fatima_abrao@yahoo.com.br (M.d.F.N.d.S.A.); cassiavitoriaalves@gmail.com (C.V.d.D.A.S.); 5 Institute of Biological Sciences, Federal University of Tocantins, Tocantins 77001-090, Brazil; 6 Public Health Laboratory of Tocantins State (LACEN/TO), Tocantins 77016-330, Brazil; 7 Postgraduate Program in Health Science, Faculty of Medicine of ABC, Santo André 09060-870, Brazil; skomninakis@yahoo.com.br; 8 Retrovirology Laboratory, Federal University of São Paulo, São Paulo 04023-062, Brazil; 9 Blood Systems Research Institute, San Francisco, CA 94143, USA; xdeng@bloodsystems.org (X.D.); eric.delwart@ucsf.edu (E.D.); 10 Department Laboratory Medicine, University of California San Francisco, San Francisco, CA 94143, USA; 11 Institute of Biological Sciences, Federal University of Pará, Pará 66075-000, Brazil

**Keywords:** saffold virus, cardiovirus, virome, picornavirus, ribosomal frameshifting, GGUUUUU motif, RNA-dependent RNA-polymerase

## Abstract

Here we report the nearly full-length genome of a recombinant Saffold virus strain (SAFV-BR-193) isolated from a child with acute gastroenteritis. Evolutionary analysis performed using all available near-full length Saffold picornavirus genomes showed that the breakpoint found in the Brazilian strain (SAFV-BR-193) is indeed a recombination hotspot. Notably, this hotspot is located just one nucleotide after the ribosomal frameshift GGUUUUU motif in the SAFV genome. Empirical studies will be necessary to determine if this motif also affects the binding affinity of RNA-dependent RNA-polymerase (RdRp) and therefore increases the changes of RdRp swap between molecules during the synthesis of viral genomes.

## 1. Introduction

The next-generation sequencing (NGS) of clinical and environmental samples has increased the identification of new species of the *Picornaviridae* family [1]. The family is divided currently into 29 genera, and the natural hosts of these viruses are vertebrates, including mammals and birds. Enteroviruses, polioviruses, hepatoviruses, and aphthoviruses are the most exhaustively characterized animal pathogens, and are associated with a wide spectrum of clinical manifestations, including undifferentiated febrile illness, respiratory illness, aseptic meningitis, and acute flaccid paralysis (AFP).

In 2007, the first Saffold virus (SAFV) was sequenced from a viral isolate generated in 1981 from the feces of an eight month-old patient with fever of unknown cause [2]. Following this initial report, numerous clinical and epidemiological studies described the worldwide prevalence of SAFV in humans from distinct regions. Although the majority of cases of SAFV infection have been described in children with gastroenteritis, respiratory tract infection and non-polio acute flaccid paralysis (AFP), there is no consensus on whether SAFV is in fact pathogenic [3,4,5]. SAFV has been classified in the genus *Cardiovirus* with positive sense RNA genomes of approximately 8000 nucleotides containing a 5’ untranslated region (UTR) with a type II internal ribosome entry site (IRES) that guide the machinery of translation to initiate synthesis of a polyprotein. This polyprotein is then processed by the viral protease into structural proteins, replicases, VPg’s, and various proteins that modify the host cells [3,6].

Encephalomyocarditis viruses (EMCV), a distinct species in the *Cardiovirus* genus, is capable through a -1 frameshift at the beginning of the 2B encoding ORF of encoding an alternative 128–129 amino acid long 2B* protein [6]. It is believe that a similar frameshift also occurs in Saffold and TMEV/RTV cardioviruses but results in only a short alternative C terminus of 14–15 amino acids. Premature termination of translation of the polyprotein produces a higher ratio of structural versus downstream encoded non-structural proteins which may enhance viral replication [6].

Phylogenetic studies performed on the *VP1* gene region of SAFV suggest that there are distinct genotypes (SAFV-1 to SAFV-11) [7,8,9]. Genotypes SAFV-1, SAFV-2 and SAFV-3 are well-defined phylogenetic clades that are globally distributed while genotypes SAFV-4 through SAFV-11 were previously found only in Afghanistan and Pakistan [1,7,8,10,11,12,13].

Here we describe and fully characterize a new recombinant SAFV strain from Brazil (SAFV-BR-193). The Brazilian strain has a unique recombination point between genes *2A* and *2B*, and it is a chimera composed of genotypes 2 and 5.

## 2. Materials and Methods

### 2.1. Sample Processing

The protocol used to perform deep-sequencing was a combination of several protocols normally applied to viral metagenomics and/or virus discovery, and has been partially described by da Costa et al. [14]. In summary, 50 mg of the human BR-193 fecal sample was diluted in 500 μL of Hanks’ buffered salt solution (HBSS), added to a 2 mL impact-resistant tube containing lysing matrix C (MP Biomedicals, Santa Ana, CA, USA) and homogenized in a FastPrep-24 5G Homogenizer (MP biomedicals, USA). The homogenized sample was centrifuged at 12,000× *g* for 10 min, and approximately 300 μL of the supernatant was then percolated through a 0.45 μm filter (Merck Millipore, Billerica, MA, USA) in order to remove eukaryotic- and bacterial-cell-sized particles. Approximately 100 μL, roughly equivalent to one fourth of the volume of the tube, of cold PEG-it Virus Precipitation Solution (System Biosciences, Palo Alto, CA, USA) was added to the obtained filtrate, and the contents of the tube were gently mixed then incubated at 4 °C for 24 h. After the incubation period, the mixture was centrifuged at 10,000× *g* for 30 min at 4 °C. Following centrifugation, the supernatant (~350 μL) was discarded. The pellet, rich in viral particles, was treated with a combination of nuclease enzymes (TURBO DNase and RNase Cocktail Enzyme Mix-Thermo Fischer Scientific, Waltham, MA, USA; Baseline-ZERO DNase-Epicentre, Madison, WI, USA; Benzonase-Darmstadt, Darmstadt, Germany; and RQ1 RNase-Free DNase and RNase A Solution-Promega, Madison, WI, USA) in order to digest unprotected nucleic acids. The resulting mixture was subsequently incubated at 37 °C for 2 h.

After incubation, viral nucleic acids were extracted using a ZR & ZR-96 Viral DNA/RNA Kit (Zymo Research, Irvine, CA, USA) according to the manufacturer’s protocol. The cDNA synthesis was performed with AMV reverse transcription reagent (Promega, WI, USA). Second strand cDNA synthesis was performed using DNA Polymerase I Large (Klenow) Fragment (Promega, WI, USA). Subsequently, a Nextera XT Sample Preparation Kit (Illumina, San Diego, CA, USA) was used to construct a DNA library, which was identified using dual barcodes. For size range, Pippin Prep (Sage Science, Inc., Beverly, MA, USA) was used to select a 300 bp insert (range 200–400 bp). The library was deep-sequenced using the Hi-Seq 2500 Sequencer (Illumina, CA, USA) with 126 bp ends. Bioinformatics analysis was performed according to the protocol previously described by Deng et al. [15]. The contigs, including sequences of rotaviruses as well as enteric viruses, humans, fungi, bacteria and others, sharing a percent nucleotide identity of 95% or less were assembled from the obtained sequence reads by *de novo* assembly. The resulting singlets and contigs were analyzed using BLASTx to search for similarity to viral proteins in GenBank’s Virus RefSeq. The contigs were compared to the GenBank nonredundant nucleotide and protein database (BLASTn and BLASTx). After the identification of a saffold virus, a reference template sequence was used for mapping the full-length genome with Geneious R9 software (Biomatters Ltd L2, Auckland, New Zealand).

### 2.2. DNA Alignment

BLASTn was initially used to identify viral sequences through their sequence similarity to annotated viral genomes in GenBank. Based on the best hits of the blastx searches, the following 46 genomes, listed by their Genbank numbers, were chosen to be used in the next analyses: AB747258, AB747257, AB747256, AB747255, AB747254, AB747252, AB747250, JN652232, JX122403, HM181996,HQ162476, FJ463615, GU943518, FJ463616, GU943513, FR682076, GU943514, FM207487, KP972594, AB983594, AB983595, HQ902242, EU681178, HM181999, EU681179, HM181997, HM181998, AB747249, EU681177, GU595289, EU376394, JF813004, AM922293, FN999911, JN652233, EU681176, JN652231, AB747253, AB747251, JX163901, EF165067, AB747248, JX122401, JX122400, JX122399, JX122402. These genomes were then aligned using Clustal X software [16]. The BR-193 strain has been deposited in GenBank under accession number MH778548.

### 2.3. Recombination Analysis

To determine the extent of recombination among sequences, we used software RDP v.4 [17], which utilizes a collection of methods. Below is a brief description of these methods; additionally, an excellent and detailed explanation of each method implemented in the RDP program can be found in the user’s manual (http://darwin.uvigo.es/rdp/rdp.html). 3Seq performs an exact nonparametric test to detect recombination in triplets of sequences. Maximum χ^2^ (MaxChi) is a method implemented by Maynard-Smith, and it uses variable/invariable sites to detect recombination in pairs of sequences. This method generates random sequence pairs; the significance level is evaluated by the proportion of simulated sequence pairs with maximum χ^2^ values higher than the real data. The maximum match χ^2^ (Chimaera) is a modification of Smith’s method. It uses variable sites to calculate the maximum χ^2^ match statistics. Geneconv detects gene conversions (recombination) by evaluating conserved substitutions in fragments between two sequences. Although evolutionary methods are not explicitly implemented in Geneconv, it is robust and has low levels of false positive detection of recombination, including those events due to rate heterogeneity and natural selection. Bootscanning is a sliding window method that was developed to identify the parental origins of sequence fragments (windows) within known or putative recombinant sequences. Lard is similar to MaxChi, and the method scans an alignment of three sequences (a recombinant and two parental sequences) for the point in the alignment that optimally separates regions of conflicting phylogenetic signals; *p*-values are also estimated to the breakpoint. Initially, we used default parameters; we later optimized the parameters in order to avoid detection of false positive recombination. In addition, window sizes of 50 to 350, stepping of 50–100 nt, as well as Bonferroni correction with *p*-values of 0.05 and 0.001 were utilized. To estimate recombination rates the INTERVAL program (within the RDP program) was used, it estimates variations in recombination rates along an alignment using a penalized approximate likelihood approach within a Bayesian reversible-jump Markov chain Monte Carlo (RJMCMC) approach. The initial estimate of the alignment-wide population scaled recombination rate (rho as a starting point) was the default and then we evaluated distinct initial values (0–30) to see if it could affect the results.

### 2.4. Phylogenetic Trees and Likelihood Mapping

Phylogenetic trees were constructed using the Maximum Likelihood approach, and branch support values were assessed using the Shimodaira-Hasegawa test. All trees were inferred using FastTree software [18]. The GTR model and gamma distribution were selected according to the likelihood ratio test (LRT) implemented in the jModeltest software [19]. Likelihood mapping was obtained using the software Tree-puzzle, version 5.3 [20], assuming GTR model and rate of heterogeneity for the evolutionary model. Analyses were performed using 1000 replications.

## 3. Results

Fecal specimens, from a survey conducted from 2010 to 2016 in the northern state of Tocantins in Brazil, were screened for the presence of enteric pathogens (i.e., rotavirus and norovirus), bacteria (i.e., *E. coli* and *Salmonella* sp.), endoparasites (i.e., *Giardia* sp.), and helminthes using conventional (i.e., culture techniques) and molecular methods (i.e., commercial enzyme immunoassays). The patient was infected by rotavirus. Following this initial screening, NGS techniques were used to identify possible undetected enteric viruses. We found a recombinant Saffold strain named SAFV-BR-193. This strain was isolated from a four-year old patient presenting acute gastroenteritis in 2015. This patient was an inhabitant of Araguaína, the second largest city in the northern state of Tocantins, which was founded in 1958 and is located 384 km from the state capital, Palmas. Its population was estimated to be approximately 175,960 in 2017 and its area is 4000.4 km^2^, with a density of 43.99 hab./km^2^. Araguaína is a humid tropical region characterized by shrub-lands, many rivers, and vast soybean plantations.

### 3.1. Polyprotein Tree

**Figure 1:**
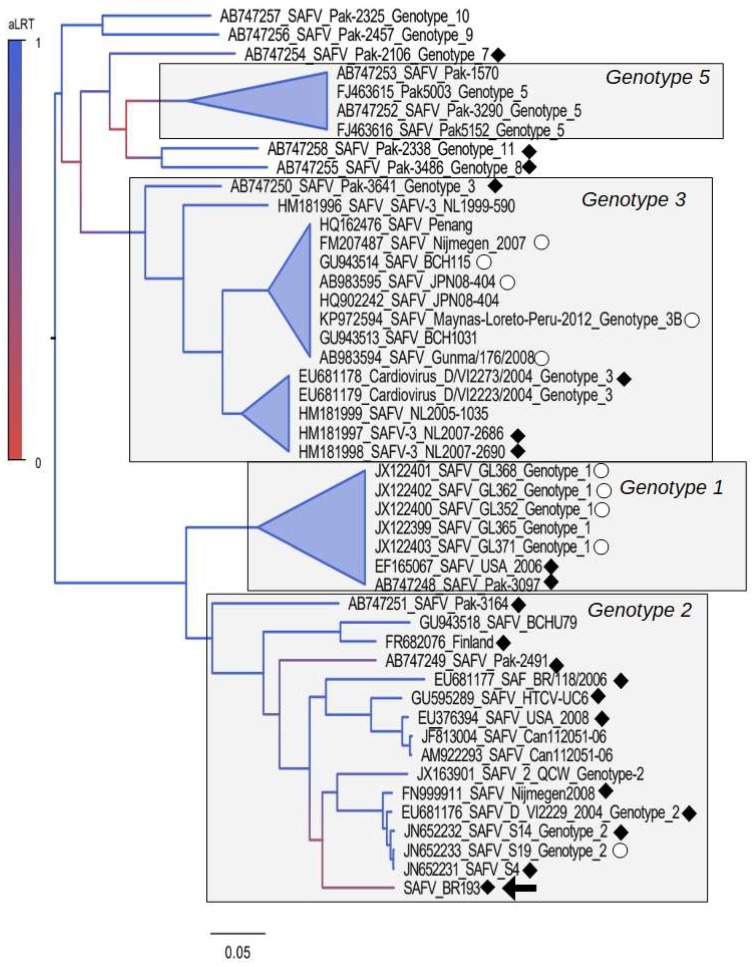
Maximum likelihood tree constructed using polyprotein region of Saffold viruses (SAFV). The Brazilian strain BR-183 described in this work is indicated by a filled arrow. A colored scale indicating the statistical support of each node, calculated using the approximate likelihood ratio test (aLRT), is shown in the tree. Phylogenetic groups corresponding to genotypes are limited by grey areas. The topology shows that genotype 1 is closely related to genotype 2, while genotype 3 is closely related to genotype 8. Genotype reference strains are indicated by open dots on the genome tree. Recombinant strains are indicated by filled diamonds in the tree. The scale bar under the tree represents the nucleotide substitutions per site.

The near-full length genome (7678 nt) of SAFV-BR-193 was sequenced and compared with the 46 previously described near-full length saffold virus genome strains; the phylogenetic tree constructed using the polyprotein region is shown in Figure 1. This tree indicates that there are at least four independent clades corresponding to genotypes 1, 2, 3, and 5 (SAFV-1, SAFV-2, SAFV-3 and SAFV-5). It also demonstrates that our isolate BR-193 is located within the clade formed by the strains of genotype 2. Note that the branch of BR-193 has low statistical support (aLRT = 0.31). For comparative purposes, we also constructed trees using the 47 near-full length genomes and the VP1 gene. All trees were nearly identical, with respect to the clustering pattern of the genotypes and the overall topology (see Figure S1 in the supplementary material).

### 3.2. Recombination Rates

**Figure 2:**
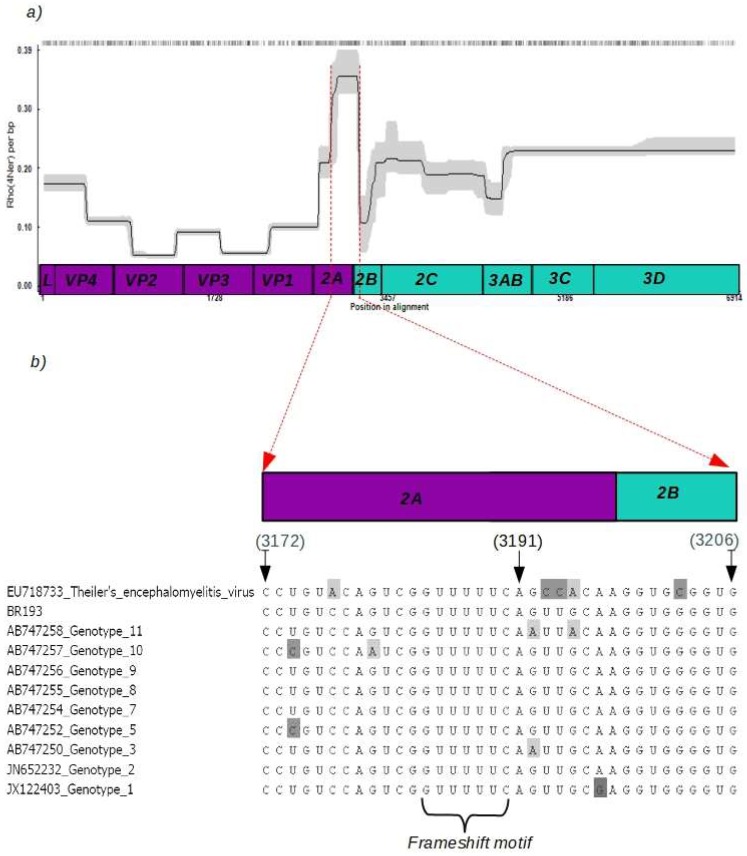
Recombination rates in the polyprotein of SAFV. The upper panel shows the recombination rates along the polyprotein of the SAFV. The dark line represents the calculated site-by-site recombination rate in the polyprotein and the gray area is the 95% credibility interval. In the x-axis there is a diagram showing the genes of the SAFV polyprotein. The genomic region corresponding to the genes *2A* and *2B* with the highest recombination rates is shown by a dashed red line the diagram (**a**). Recombination rates were estimated using INTERVAL program in the RDP. The lower panel shows the region between genes *2A* and *2B* to indicating the conserved GGUUUUU motif in an alignment of SAFV strains. The diagram above the alignment indicates the location (nucleotides 3172 to 3206) of the motif in the SAFV polyprotein (**b**).

Previous studies have shown that low values of branch support in maximum likelihood trees, besides rate of heterogeneity, may also indicate recombination events [12,21]. For this reason, we performed a detailed analysis (summarized in Figure 2) in order to determine the extent of recombination in the SAFV polyprotein region. Figure 2 shows a peak in recombination in a region (nucleotides 3172–3206) that corresponds to the limit between genes *2A* and *2B* (upper panel in Figure 2). Notably, this region also includes the canonical GGUUUUU motif used by cardioviruses as a −1 nt ribosomal frameshift (middle panel in Figure 2). In fact, since the GGUUUUU motif has the theoretical propensity to form loop structure (lower panel in Figure 2), it may likely interfere with RdRp binding affinity to RNA and, therefore, increase changes in molecule swaps during synthesis.

### 3.3. Partitioned Trees

**Figure 3:**
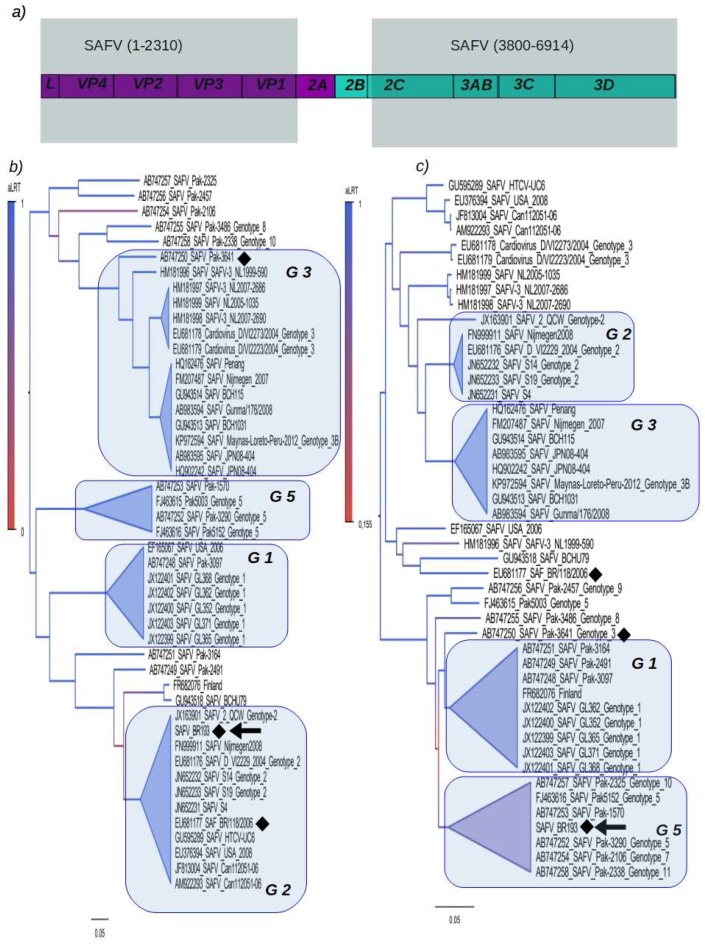
Trees inferred with the partitioned SAFV polyprotein. The polyprotein alignment was divided in two partitions and the region corresponding to the genes *2A* and *2B* was excluded (**a**). Each partition was used to construct a maximum likelihood (ML) tree. The ML tree inferred using the partition corresponding to nucleotides 1 to 2310 of the SAFV polyprotein shows clusters of sequences corresponding to the main SAFV genotypes (**b**). The ML tree inferred using the partition corresponding to nucleotides 3800 to 6914 of the SAFV polyprotein equally shows clusters corresponding to the main genotypes (**c**). In this topology genotypes 2 and 3 are the closest. Both trees show that some isolates (mainly from Pakistan) are not included in cluster and they locate at the base of different genotypes. Recombinant strains are indicated by filled diamonds and the BR-193 strains is indicated by an arrow. Trees were mid rooted and strains clustering within certain clades, corresponding to SAFV genotypes (indicated by blue colored areas), were collapsed in the tree (blue triangles). The vertical scale bar represents the statistical support for branches was estimated using the approximate likelihood ratio test (aLRT). Horizontal bars represent the amount of nucleotides substitution per site.

Next, we characterized in detail the recombination pattern of our isolate SAFV-BR-193. In order to do so, the genomic region corresponding to genes *2A* and *2B* was excluded because of its high recombination rates (upper panel of Figure 3). The polyprotein alignment was then partitioned into genomic regions 1 to 2310 and 3800 to 6914 (shaded areas in the upper panel of Figure 3). These two partitions were used to infer trees. The tree constructed with the partition 1 to 2310 (panel b in Figure 3) showed the isolate SAFV-BR-193 within the clade formed by strains of genotype 2, while the tree constructed with the partition 3800 to 6914 showed SAFV-BR-193 in the clade of genotype 5 (panel c in Figure 3). Nucleotides 1 to 3910 of the Brazilian SAFV-BR-193 is related to the strain SAFV-S4 (JN652231) of genotype 2 and both diverged in only 10%. Similarly, the genomic region 3910 to 7678 of SAFV-BR-193 is related to the strain Pak-3290 (AB747253) of genotype 5 and they diverged by 7%. It is also interesting to note that many isolates from Pakistan (named Pak) are located in different clusters in both trees. For example, the isolate Pak-3641 (AB747250) was located within the clade of genotype 3 in the tree corresponding to the region 1 to 2310, while in the tree corresponding to the region 3800 to 6914, this isolate was located at the base of the cluster of genotype 1 (indicated in Figure 3 by a filled diamond). For illustrative purposes, we have also described the isolate BR118/2006, which is a mosaic composed of genotypes 2 and 3 (see Figure S4 in the supplementary material). We used pairwise distances and likelihood mapping to illustrate the evolutionary differences in the SAFV polyprotein. We noticed that the genetic distances among strains are higher in the partition corresponding to nucleotides 1 through 2310 than the distances in the partition corresponding to nucleotide 3800 through 6914. Likewise, the phylogenetic signal (measured by the amount of unresolved quartet trees, see Figure S2 in the supplementary material) is better in the partition corresponding to nucleotides 1 through 2310.

### 3.4. Mosaic Pattern

**Figure 4:**
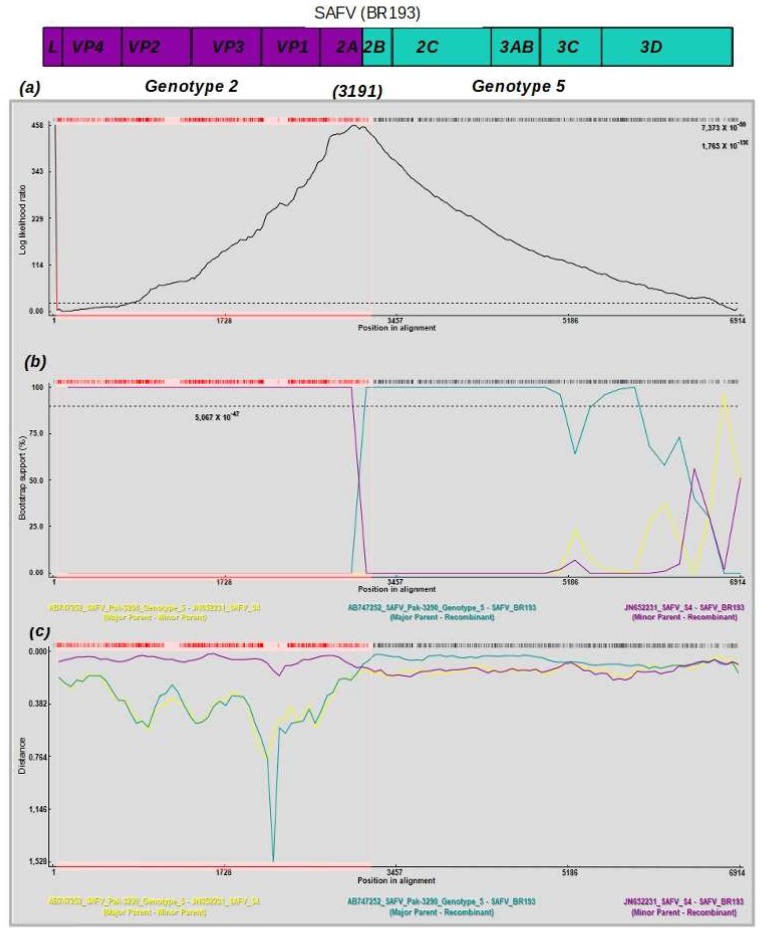
Recombination pattern of SAFV BR193. The upper panel shows the position of the recombination breakpoint in the polyprotein of SAFV (**a**). This is based on the LARD method that detects recombination breakpoints by scanning an alignment of three sequences (one recombinant and two putative parental sequences) for the point (likelihood ratio test) in the alignment that optimally separates regions of conflicting phylogenetic signal. The plot (solid black line) indicates the point of maximum likelihood and the values above the peak are the approximate *p*-value. There are two *p*-values calculated (upper right in the plot). The upper value corresponds to a breakpoint detected at position 63, this peak was not confirmed by other methods. The second value in the upper right of the plot correspond to the breakpoint at the position 3191 of the polyprotein. The upper dashed black and red lines indicate partitions in the alignment that presented conflicting trees. Dashed lines indicate the cutoff value based on Bonferroni correction test. The vertical dashed arrow indicates the breakpoint position in the SAFV polyprotein. The bootscanning method was used to determine the parental genotypes that compose the recombinant strain BR193 (**b**). Colored lines represent the probability (given in bootstrap value) of genomic regions to belong to a certain parental genotype. The x-axis represents the sequence length in base pairs (bp). The y-axis represents the statistical support (bootstrap) based on 500 replicates. Each plotted line refers to a certain genotype (see the sequence code color below the x-axis). The plot indicates a single breakpoint in the polyprotein region of the isolate BR-193 at the position 3191. The evolutionary model Felsenstein, 1984 plus the estimated transition/transversions (ts/tv = 3.62) were used. Window sizes of 50 to 350, stepping of 50–100 nt, as well as χ^2^ correction with *p*-values of 0.05 and 0.001 were utilized. Pairwise distances were used to determine the proximity of BR-193 to other strains (**c**). The colored lines show the distances between the isolate BRTO83 and the references, and the lower panel shows the distances between BR-193 and the SAFV references. The y-axis indicates the genetic distances, and the x-axis shows the nucleotide positions in the SAFV polyprotein. All these analyses were performed using the RDP v4 software [17].

We also determined the mosaic pattern and the position of the recombination breakpoint of the isolates SAFV-BR-193 and Pak-3641. In order to do this, the polyprotein was used (Figure 4 and supplementary material). Initially, we used a method (i.e., Lard) that combined different triplet sequences to determine the positions of breakpoints and to provide *p*-values for these positions in the alignment (see Materials and Methods for a detailed description). In both isolates, only one breakpoint was detected. The recombination breakpoint detected in isolate SAFV-BR-193 is located at position 3191 of the polyprotein (corresponding to position 3910 of the full genome, upper panel in Figure 4); in isolate Pak-3641, it was located in position 2802 (corresponding to position 3821 of the full genome, upper panel in the supplementary Figure S3). Next, two methods based on sliding windows (i.e., bootscanning and similarity plot) were used to show the similarity and parental relatedness of the recombinant isolates BR-193 and Pak-3641 (middle and lower panels in Figure 4 and Figure S3 in the supplementary material). Concerning isolate BR-193, nucleotides 1 through 3191 are related to isolate JN652231 of genotype 2, and the remaining nucleotides (3192 to 6914) are related to isolate AB747252 of genotype 5. Similarly, the first 2802 nucleotides of isolate Pak-3641 appeared similar to isolate GU943814 of genotype 3, and nucleotides 2803 to 6914 appeared similar to isolate AB747252 of genotype 5. Similarly, isolate BR118/2006 (EU68117) has a single breakpoint at position 2761 of its polyprotein and is a mosaic composed of genotypes 2 and 3 (see supplementary Figure S4). These results are in agreement with the partitioned trees that show isolates BR-193 and Pak-3641 in distinct clades.

## 4. Discussion

The family *Picornaviridae* contains many pathogens such as poliovirus, coxsackievirus, rhinovirus, parechovirus, and cardiovirus [1,9]. Recently, another species in the genus cardiovirus was isolated from humans and was named Saffold virus (SAFV) [2,22]. Although the connection between human disease and SAFV infection has not been completely elucidated, there is growing evidence for the involvement of SAFV in acute respiratory infections and inflammation of the myocardium [4,13]. Epidemiological surveys indicate that SAFV is spread worldwide, infecting mainly young children. Additionally, it has been suggested that SAFV can been classified as eleven different genotypes (SAFV-1 to SAFV-11) [1,8,23].

Here we describe a nearly-complete genome of an SAFV strain (named BR-193) from Brazil that was isolated from a child presenting acute gastroenteritis, likely caused by a rotavirus co-infection. The BR-193 strain is a recombinant between genotypes 2 and 5. Interestingly, genotypes SAFV-5 through SAFV-11 have been isolated primarily from individuals from Pakistan and Afghanistan, while SAFV-2 and SAFV-3 have been observed in individuals from America, Europe, and Asia [1,10,24]. This is a result of the limited number of complete genomes available, since we found recombinants between parental genotypes that were not yet identified in the same geographic regions (viz., genotypes 2 and 5). The phylogenies constructed using two partitions of the polyprotein also indicate that some isolates of genotypes SAFV-5 through SAFV-11 may be recombinants because they cluster in distinct groups in each tree. Another feature of SAFV demonstrated by this study is that there is a recombination hotspot between genes *2A* and *2B*. There is a conserved motif, GGUUUUU, in the beginning of the ORF corresponding to *2B*. In EMCV, this motif is involved in generating a ribosomal frameshift resulting in expression of an alternative 2B* protein and the same phenomenon may occur in Saffold viruses but resulting in a shorter trans-frame fusion peptide [6]. We speculate that the GGUUUUU motif followed 14–15 nucleotides downstream by a potential RNA stem-loop may also affect the processivity of the RdRp and thus increase the possibility of RNA template swapping during RNA synthesis resulting in viral recombination. Further studies will be necessary to determine if the recombination hotspot between 2A and 2B is influenced by the same structures involved in ribosome frameshifting.

## Supplementary Materials

The following are available online at http://www.mdpi.com/1999-4915/10/10/520/s1, Figure S1: Genome tree of SAFV, Figure S2: Phylogenetic signal in the polyprotein of SAFV, Figure S3: Mosaic pattern of SAFV-Pak3641, Figure S4: Mosaic pattern of SAFV-BR118/2006.
