# Supplementary material for: Recombination Located over 2A-2B Junction Ribosome Frameshifting Region of Saffold Cardiovirus
Source: Viruses. 2018 Sep 24;10(10):520. doi: 10.3390/v10100520 (PMC6213509; doi:10.3390/v10100520)
Supplement: Supplementary file 1 [file viruses-10-00520-s001.pdf]

Figure 1. Maximum likelihood trees of SAFV

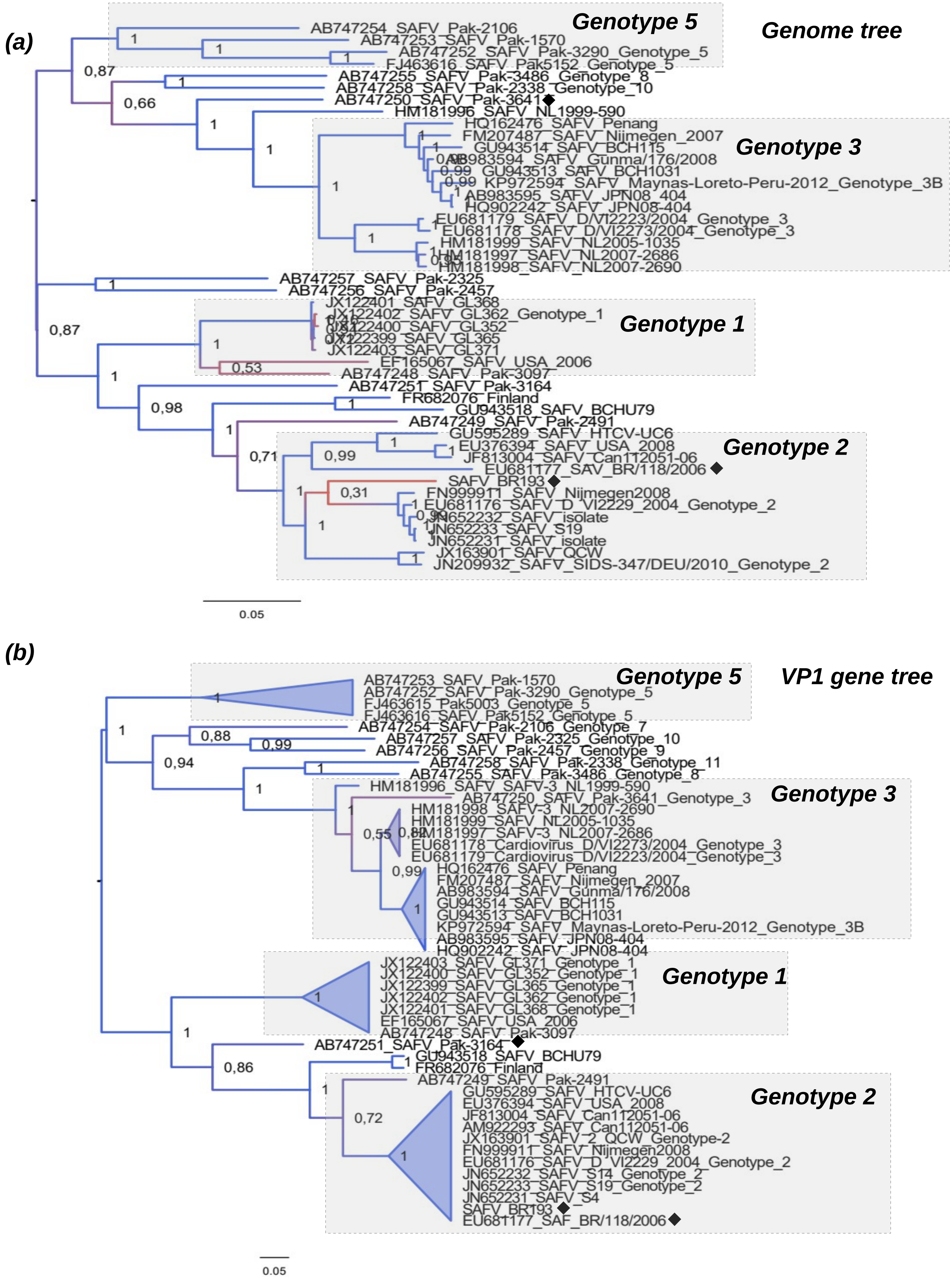

Figure 1. Phylogenetic tree of SAFV

Maximum likelihood tree constructed with near full length genomes (a) and VP1 gene of SAFV (b). Values on the tree indicate the statistical support of each node that were calculated using the approximate likelihood ratio test (aLRT). A colored scale indicating the aLRT is also shown in the trees. Phylogenetic groups corresponding to genotypes are limited by grey areas. Recombinant strains were indicated by filled diamonds in the tree. Scale bar under the tree represent the nucleotide substitution per site. Trees were constructed using maximum likelihood criteria implemented in the FastTree software [1], assuming GTR model plus gamma correction distributions and the proportions of variable sites in the alignment.

Figure 2. Phylogenetic signal in the polyprotein of SAFV

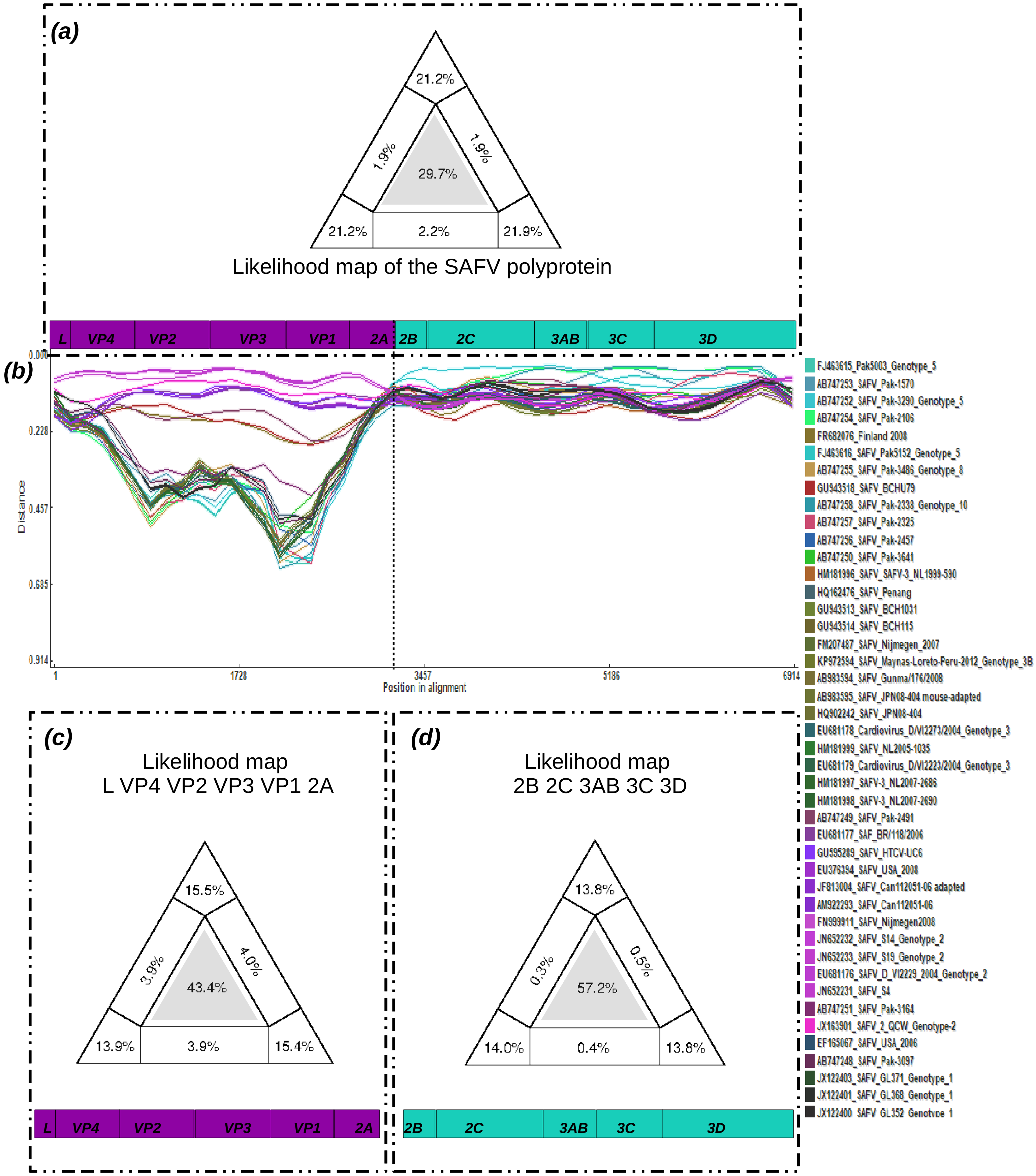

Figure 2. Phylogenetic of SAFV polyprotein.

The upper panel show the likelihood map constructed using the polyprotein alignment with 47 SAFV isolates (a). The lower panel also shows likelihood maps of the partition corresponding to genes L VP4 VP2 VP3 VP1 2A of the polyproteins (c) and the partition corresponding to the genes 2B 2C 3AB 3C 3D of the SAFV polyprotein (d). The likelihood mapping summarizes the likelihoods of quartet trees in a triangular diagram that represent the percentages of all possible quartet-trees in the alignment[2]. Vertices represent fully resolved quartets and the central area of the triangles (triangular gray area) indicates the amount of unresolved trees (star trees). The areas connecting vertices represent partially resolved trees. The higher is the percentage of star trees the lower if the phylogenetic signal of the alignment. The quartet-mapping analysis were made using the software TreePuzzle v 5.3[3] assuming the GTR model plus gamma correction and the proportion of invariable sites.

The middle panel is a distance plot that shows the pairwise distances among all 47 SAFV isolates are also show in the distance plot (b). Each colored line represent the site-by-site distance between BR-193 and one SAFV strain (see the color code in the left side of the figure). The Y-axis indicate the genetic distances and the x-axis shows the nucleotide positions in the SAFV polyprotein. The evolutionary model Fenselstein, 1984 plus the estimated transition/transversions (ts/tv=3,62) were used. Window sizes of 50 to 350, stepping of 50-100 nt, as well as $\chi 2$ correction with p-values of 0.05 and 0.001 were utilized. The vertical dashed arrow indicate the breakpoint position in the SAFV polyprotein. All these analysies were perfomed using the RDP v4 software[4].

Figure 3. Recombination pattern of SAFV Pak3641

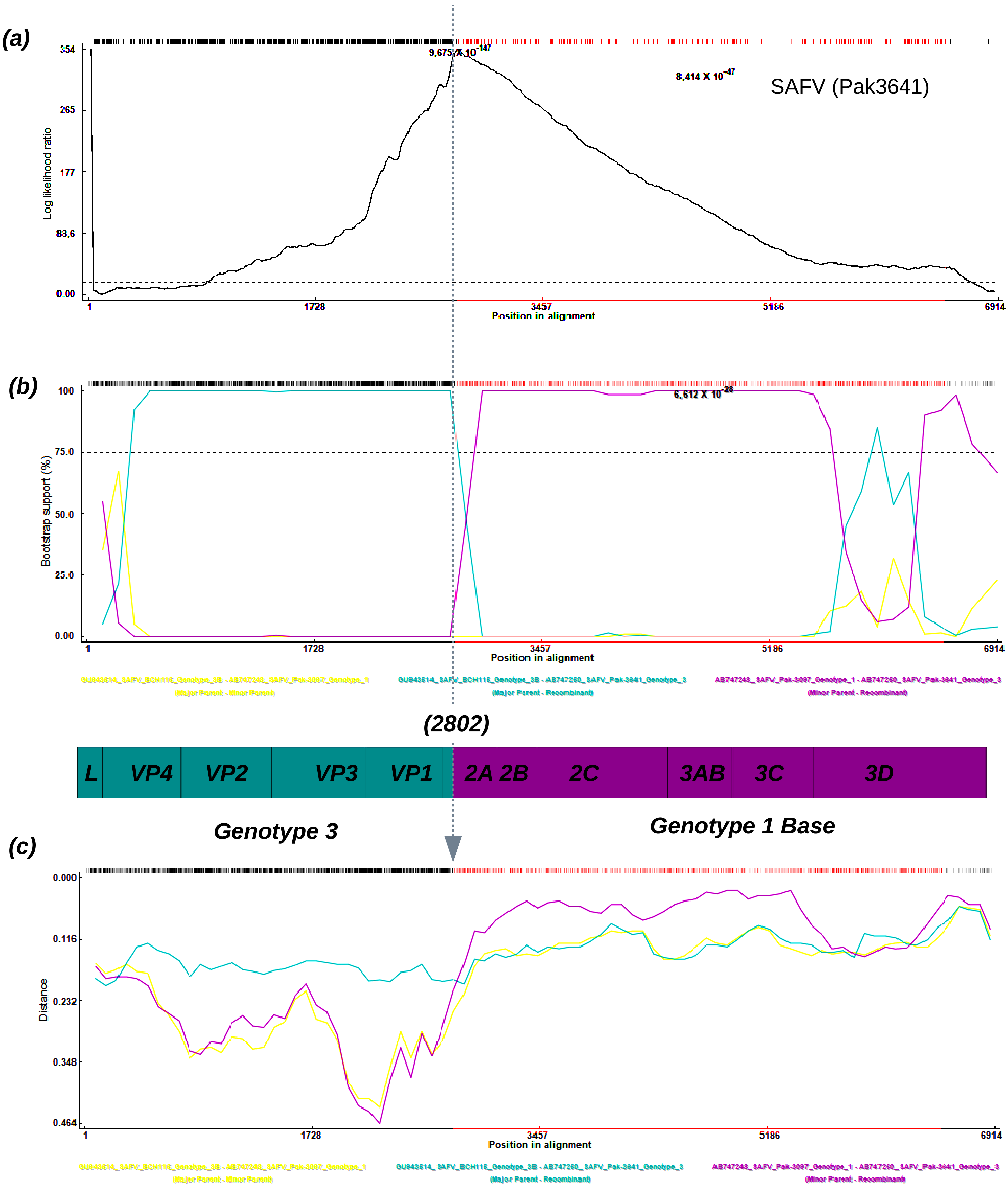

Figure 3. Mosaic pattern of SAFV-Pak3641.

The position of the recombination breakpoint in the polyprotein of SAFV-Pak3641 (a). The plot (solid black line) indicate the point of maximum likelihood and the values above the peak is the approximate p-value. The recombination breakpoint at the position 2802 of the SAFV polyprotein. The upper dashed black and red lines indicate partitions in the alignment that presented conflicting trees. Dark and light gray are is the 95% confidence interval for breakpoint position. Dashed line indicate the cutoff value based on Bonferroni correction test. The vertical dashed arrow indicate the breakpoint position in the SAFV polyprotein. The bootscanning method was used to determine the parental genotypes that compose the recombinant strain SAFV-Pak3641 (b). Colored lines represent the probability (given in bootstrap value) of genomic regions to belong to a certain parental genotype (i.e., getotype 3 and genotype 1). The x-axis represents the sequence length in base pairs (bp). The y-axis represents the statistical support (bootstrap) based on 500 replicates. Each plotted line refers to a certain genotype (see the sequence code color below the x-axis). The plot indicate a single breakpoint in the polyprotein region of the isolate BR-193 at the position 3191. The evolutionary model Fenselstein, 1984 plus the estimated transition/transversions (ts/tv=3,62) were used. Window sizes of 50 to 350, stepping of 50-100 nt, as well as χ^2 correction with p-values of 0.05 and 0.001 were utilized. The vertical dashed arrow indicate the breakpoint position in the SAFV polyprotein.

Pairwise distance were used to determine the proximity of SAFV-Pak3641 with other strains (c). The colored lines shows the distances between the isolate SAFV-Pak3641 and the references and lower shows the distances between BR-193 and the SAFV references. The Y-axis indicate the genetic distances and the x-axis shows the nucleotide positions in the SAFV polyprotein.

Figure 4. Recombination pattern of SAFV BR118/2006

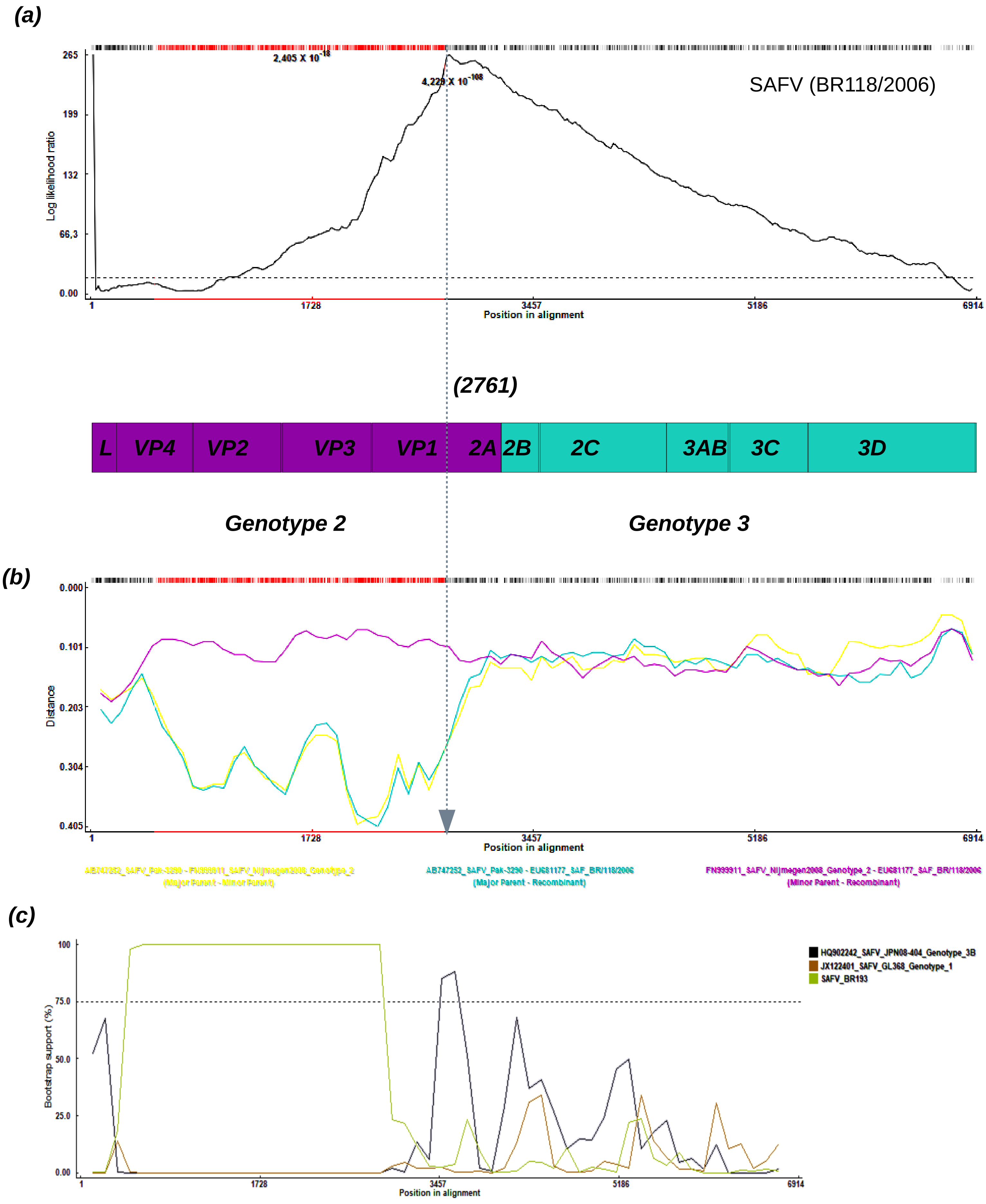

Figure 4. Mosaic pattern of SAFV-BR118/2006.

The position of the recombination breakpoint in the polyprotein of SAFV-BR118/2006 (a). The plot (solid black line) indicate the point of maximum likelihood and the values above the peak is the approximate p-value. The recombination breakpoint at the position 2761 of the SAFV polyprotein. The upper dashed black and red lines indicate partitions in the alignment that presented conflicting trees. Dark and light gray are is the 95% confidence interval for breakpoint position. Dashed line indicate the cutoff value based on Bonferroni correction test. The vertical dashed arrow indicate the breakpoint position in the SAFV polyprotein. The bootscanning method was used to determine the parental genotypes that compose the recombinant strain SAFV-BR118/2006 (b). Colored lines represent the probability (given in bootstrap value) of genomic regions to belong to a certain parental genotype (i.e., getotype 3 and genotype 1). The x-axis represents the sequence length in base pairs (bp). The y-axis represents the statistical support (bootstrap) based on 500 replicates. Each plotted line refers to a certain genotype (see the sequence code color below the x-axis). The plot indicate a single breakpoint in the polyprotein region of the isolate SAFV-BR118/2006 at the position 2761. The evolutionary model Fenselstein, 1984 plus the estimated transition/transversions (ts/tv=3,62) were used. Window sizes of 50 to 350, stepping of 50-100 nt, as well as χ^2 correction with p-values of 0.05 and 0.001 were utilized. The vertical dashed arrow indicate the breakpoint position in the SAFV polyprotein.

Pairwise distance were used to determine the proximity of SAFV-Pak3641 with other strains (c). The colored lines shows the distances between the isolate SAFV-BR118/2006 and the references: BR-193, JPN08-404 (genotype 3) and GL368 (genotype 1). This comparison was made to show that SAFV-BR118/2006 and BR-193 are not identical although they circulalete in the same country.

The Y-axis indicate the genetic distances and the x-axis shows the nucleotide positions in the SAFV polyprotein. The evolutionary model Fenselstein, 1984 plus the estimated transition/transversions (ts/tv=3,62) were used. Window sizes of 50 to 350, stepping of 50-100 nt, as well as χ^2 correction with p-values of 0.05 and 0.001 were utilized. The vertical dashed arrow indicate the breakpoint position in the SAFV polyprotein. All these above recombination analysis were performed using the RDP v4 software.

References:

1 Price, M. N., Dehal, P. S. & Arkin, A. P. FastTree 2--approximately maximum-likelihood trees for large alignments. PLoS One 5, e9490, doi:10.1371/journal.pone.0009490 (2010).

2 Strimmer, K. & von Haeseler, A. Likelihood-mapping: a simple method to visualize phylogenetic content of a sequence alignment. Proc Natl Acad Sci U S A 94, 6815-6819 (1997).

3 Schmidt, H. A., Strimmer, K., Vingron, M. & von Haeseler, A. TREE-PUZZLE: maximum likelihood phylogenetic analysis using quartets and parallel computing. Bioinformatics 18, 502-504 (2002).

4 Martin, D. P., Murrell, B., Golden, M., Khoosal, A. & Muhire, B. RDP4: Detection and analysis of recombination patterns in virus genomes. Virus Evol 1, vev003, doi:10.1093/ve/vev003 (2015).
